# Study of Blood-transfusion Services in Maharashtra and Gujarat States, India

**DOI:** 10.3329/jhpn.v27i2.3368

**Published:** 2009-04

**Authors:** K.V. Ramani, Dileep V. Mavalankar, Dipti Govil

**Affiliations:** Centre for Management of Health Services, Indian Institute of Management, Vastrapur, Ahmedabad 380 015, India

**Keywords:** Blood-banks, Blood-storage centres, Blood transfusion, Emergency obstetric care, India

## Abstract

Blood-transfusion services are vital to maternal health because haemorrhage and anaemia are major causes of maternal death in South Asia. Unfortunately, due to continued governmental negligence, blood-transfusion services in India are a highly-fragmented mix of competing independent and hospital-based blood-banks, serving the needs of urban populations. This paper aims to understand the existing systems of blood-transfusion services in India focusing on Maharashtra and Gujarat states. A mix of methodologies, including literature review (including government documents), analysis of management information system data, and interviews with key officials was used. Results of analysis showed that there are many managerial challenges in blood-transfusion services, which calls for strengthening the planning and monitoring of these services. Maharashtra provides a good model for improvement. Unless this is done, access to blood in rural areas may remain poor.

## INTRODUCTION

Blood is a vital healthcare resource used in a broad range of hospital procedures, viz. accidents, emergency obstetric services, and other surgeries. It is also a potential vector for harmful, and sometimes fatal, infectious diseases, such as HIV, hepatitis B and C. Every year, millions of people are exposed to avoidable, life-threatening risks through the transfusion of unsafe blood. As per a global database, 6 million of 81 million units of blood collected annually in 178 countries are not screened for transfusion-transmissible infections (1).

Shortfalls in supply of safe blood have a particular impact on women with pregnancy and delivery-related complications and with severe life-threatening anaemia. Worldwide, more than half a million women die each year during childbirth or in the postpartum period (2). Over 90% of maternal deaths occur in Asia and sub-Saharan Africa, with India alone accounting for 20% of such deaths (3). Severe bleeding during delivery or after childbirth is the most common cause of maternal mortality globally (25%) and contributes to around 31% of maternal deaths in Asia (2).

Because of the unpredictable nature of postpartum bleeding, blood transfusion has been identified as one of eight key life-saving functions that should be available in healthcare facilities providing comprehensive emergency obstetric care (EmOC) (2,4). Access to a safe and sufficient blood supply could help prevent deaths of a significant number of mothers and their newborn children. As per estimates globally, each year up to 150,000 pregnancy-related deaths could be avoided through access to safe blood (5).

Results of a study of the circumstances for maternal deaths in Indonesia indicate that the lack of blood supplies and inability of some healthcare professionals to administer a transfusion contributed to deaths due to haemorrhage (6). Similarly, estimates from Latin American and Caribbean countries show that the limited availability of blood for transfusion in countries with high ratios of maternal mortality may hinder comprehensive care of mothers (4). Also, the supply of blood to obstetric facilities was one of the central topics of the clinical audit of maternal deaths in Morocco. However, the effectiveness of this audit process was hindered due to poor record-keeping of availability or otherwise of blood and blood products in obstetric cases (7).

In India alone, according to a review of the Sample Registration Survey (1997-2003), postpartum haemorrhage accounts for nearly 38% of all maternal deaths (8); this is more than the Indian Council of Medical Research's estimate of 25,000 deaths every year (9). As more than half of the women of reproductive age are mild to severely anaemic (10), they are very vulnerable to dying from bleeding. As the majority (65%) of births take place at home (in some areas, it is almost 92%), and a large proportion are assisted by unskilled personnel (3), women experiencing life-threatening complications may not receive the required life-saving emergency services because of the ‘four delays'. These delays can result in maternal mortality or increased severity of morbidity. These four delays include recognizing the problem, deciding to seek care and reaching the health facility, plus the delay in receiving adequate treatment once a woman has arrived at the health facility (3). Ensuring that the health facility is adequately equipped and supplied for blood transfusion would decrease this last delay. However, a search through the PubMed and Google Scholar databases showed that the organization and management of blood-transfusion services in India has received little attention (11-15).

This global scenario is evidenced from a statement by Director General of the World Health Organization made on the World Health Day 2000. He said, “Despite all the technological marvels that humani-ty is experiencing, a reliable and safe blood supply is still out of the reach for untold millions of people around the world”.

To achieve Millennium Development Goal 5, access to effective EmOC, along with provision of professional care at birth, has been identified as vital for reduction in maternal mortality (16). In India, First Referral Units (FRUs) have been identified to deliver EmOC services. The FRU guidelines indicate that a blood-storage facility is one of the three critical determinants of FRU functionality (17) and for effective EmOC services. However, the FRUs were not fully operational due to lack of specialist staff, infrastructure, equipment, medicines, and blood-transfusion facilities in 2001 (18). It is estimated that if the FRUs were equipped with a proper blood supply, they could reduce maternal mortality by 30% (19).

**Fig. 1.**
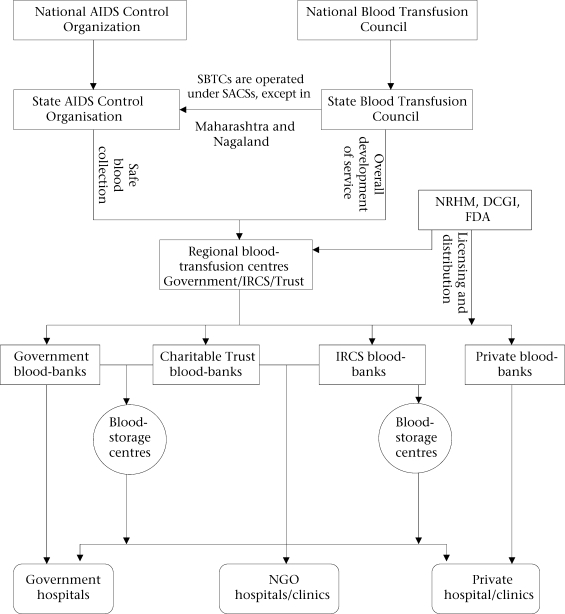
Role of organizations in blood-transfusion services in India

The primary responsibility for blood supply for blood-storage centres at the FRUs lies with the government/national health authority. Regional Blood Transfusion Centres (RBTCs), identified based on the amount of blood collected (more than 15,000 units per year), carries out this responsibility. Licensing and monitoring of the blood-banks in India is the responsibility of the Drug Controller of India. The National Blood Transfusion Council (NBTC), established in accordance with the agreed national blood policy and within a legislative framework, has the major advisory role in the formulation of policy on safe blood-transfusion services in India. The National AIDS Control Society is responsible for collection of safe blood while providing financial assistance (Fig. 1).

DCGI=Drug Controller General of India; FDA=Food and Drug Administration; IRCS=Indian Red Cross Society; NGO=Non-governmental organization; NRHM=National Rural Health Mission; SACS=State AIDS Control Organization; SBTC=State Blood Transfusion Council

Many states in India have started addressing issues concerning the absence of blood-storage centres and lack of blood in the periphery for averting maternal deaths. Some states, i.e. Tamil Nadu and Rajasthan, have launched projects independently or under the Reproductive and Child Health Plan Implementation Programme to strengthen the FRUs by setting up blood-storage centres, along with providing adequate training to blood-bank staff and doctors (19,20). Apart from being an essential determinant of EmOC services, blood transfusion is an indispensable part of abortion services (21).

The objective of this paper is to analyze the existing systems of blood-transfusion services in India, specifically focusing on the Maharashtra and Gujarat states. We have identified the managerial issues and provided recommendations for the improvement of blood-transfusion services to ensure the supply of blood to the FRUs and saving mothers.

## MATERIALS AND METHODS

Methodologies used in the paper included review of documents, reports, and other literature and analyses of the organizational structure and process of blood-banking services, specifically the State Blood Transfusion Councils (SBTCs) and State AIDS Control Societies (SACSs) in Maharashtra and Gujarat. The policy guidelines of the National AIDS Control Organization (NACO) have also been reviewed to understand the role of NACO vis-à-vis NBTC. We have compared blood-banking management in India with the framework proposed by the World Health Organization (WHO) (1). Statistics on blood-banking indicators were obtained from the respective SBTCs for 2005. Interviews with key officials of the Councils and the key staff of selected blood-banks were carried out. We also participated in meetings of stakeholders involved in blood-transfusion services.

## EVOLUTION OF THE BLOOD SAFETY PROGRAMME IN INDIA

The Blood Safety Programme in India began to take shape in 1987 with the establishment of the NACO, a government organization to oversee. In 1992, the Drug Controller General, India, was vested with the power of Central License Approving Authority to approve licensing of notified drugs, viz. blood and blood products. During the same year, under the National AIDS Control Programme I (NACP I, 1992-1999), the NACO launched a scheme to modernize blood-banks by providing assistance to states to upgrade and provide minimum facilities to blood-banks in the public sector and those run by charitable organizations, such as Indian Red Cross Society. Under the NACP I, the NACO modernized 815 blood-banks (282 major blood-banks and 533 district-level blood-banks) and also set up 40 blood component separation facilities to promote the rational use of blood. [Blood collection in major blood-banks is between 5,000 and 10,000 units and in the district-level blood-banks between 3,000 and 5,000 units per year]. Nearly 90% of the blood-banks modernized through this scheme were in the government sector. Meanwhile, in 1996, as a result of a case—Common Cause vs Union of India and others—the Supreme Court of India passed an order directing the Government to improve blood-transfusion services. Consequently, the NBTC and SBTCs were created to develop policies and programmes for improving blood-transfusion services in India.

The Blood Safety Programme initiated in the NACP I was considerably strengthened in the NACP II (1999-2004) with modernizing more blood-banks, establishing model blood-banks, and setting up blood-storage centres in rural areas. The Government of India adopted the National Blood Policy in 2002 which aimed at ensuring easy accessibility to and adequate supply of safe and quality blood and blood components collected from voluntary and non-remunerated blood donors. In India, different organizations play different roles in licensing, controlling, and regulating blood-banks. While the Drug Controller General is the licensing authority, the NACO and SACSs aim at preventing the transmission of HIV and ensure 100% screening of all collected blood units, providing training and financial assistance to blood-banks for technical modernization for ensuring the quality of blood-banks. The NBTC and SBTCs aim at ensuring safe and quality blood for transfusion services, promote voluntary and non-remunerated blood donors, follow up quality-control assurance programmes in blood-banks, and conduct training programmes. The Council collects funds by 100% tax-free donations.

**Fig. 2.**
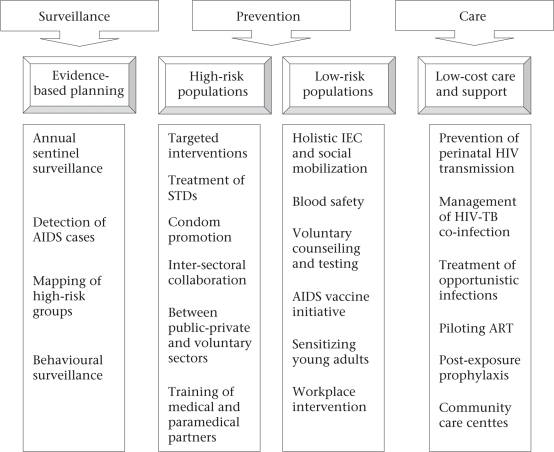
Components of the Second National AIDS Control Programme (1999-2006)

Source: National AIDS Control Society. Annual report, 2002-2004 (22) AIDS=Acquired immunodeficiency syndrome; ART=Antiretroviral therapy; IEC=Information, education, and communication; STD=Sexually transmitted diseases; TB=Tuberculosis

Despite a directive of the Supreme Court regarding formation and functioning of SBTCs, all SBTCs (except in Maharashtra and Nagaland) are functioning under the SACSs. Since the SACSs have many other responsibilities relating to AIDS control (22), blood safety and availability functions may be overlooked (Fig. 2), hence the assistance of SBTCs is necessary.

Over the years, a fragmented mix of competing independent and hospital-based blood-banks of different levels of sophistication, serving different types of hospitals and patients, have emerged. Besides the government blood-banks, there are blood-banks run by charitable trusts, independent commercially-oriented private organizations, and specific non-government health organizations, such as the Indian Red Cross Societies (IRCSs). The piecemeal evolution of blood-banking is linked to the burgeoning population and the expanding and poorly-regulated private healthcare market. Most blood-banks in public hospitals often operate with minimal infrastructure, lack of leadership, and resources; this has resulted in an inadequate/irregular supply of blood in the public system. [In India, public bodies concerned with the organization and administration of blood services include Central, State, and autonomous government institutes, municipal corporations, cantonment boards, railway services, employee state insurance authorities, and the armed forces].

## MANAGEMENT OF BLOOD-BANKS

Blood-bank activities include recruitment and retention of donors; collection, testing, processing, and storage of blood; and clinical use of blood and blood components and training (1). Review of literature and interviews with key stakeholders indicate that there are many management problems within blood-banking services. Some major issues facing the management of blood-banks in the Maharashtra and Gujarat states are described below, and a comparison of statistics on blood-banking services is presented in the table.

### Organization of blood-transfusion services

Following the Supreme Court intervention, the State Government of Maharashtra established its Blood Transfusion Council in 1996 as an independent entity separate from the Maharashtra SACS. The SBTC has a full-time professional of the rank of Assistant Director, supported by a research officer, statistical assistant, and MIS (management information system) staff. It has an objective to coordinate the fragmented blood-supply system while providing adequate, safe, and affordable blood units and components. Given its autonomous status and administrative staff, the SBTC maintains good coordination with other blood-safety units in Maharashtra, such as SACS and international development agencies. It involves the entire health infrastructure, primary hospitals to tertiary hospitals, in blood-donation campaigns as each health facility is given an annual target for blood-donation camps.

The organizational structure of the Maharashtra SBTC extends to the regional and district levels. Maharashtra has eight regional blood-transfusion officers and 34 district blood-transfusion officers. The Council has also established the following committees to improve various aspects of blood transfusion in the state: (a) Technical Committee, (b) Voluntary Blood-donation Committee, (c) Thalassaemia Committee, (d) Post-graduation Committee, and (e) Project Formulation Committee.

Many other state governments, including Gujarat, established its SBTC as a part of SACS and appointed the Additional Director of SACS as head of SBTC. Thus, the SBTC is not an independent unit and works under the head of SACS. In Gujarat, the SBTC does not have any full-time professional and administrative staff. The Council is mainly composed of honourary members and is headed by the health secretary. As the members and chairperson have many other responsibilities, the meetings of the Council are not held regularly. Compared to Maharashtra, Gujarat lacks systematic organization of the blood-transfusion council.

### Status of blood-banks

In India, there are 2,063 licensed blood-banks. A significant portion of blood-banking activity is carried out by voluntary (n=257) and private blood-banks (n=980). The total recorded blood collection in India is four million units, which meet only 40% of need against a least requirement of 10 million units (calculated at 1% of 1 billion population) [If 1-3% of a country's population donate blood, it would be sufficient for the country's needs for a year (23). The WHO bases its estimates on the number of ‘acute beds' in a given district, which depends on the type of medical services available and the location of a health facility]. However, as per estimates done by the Gujarat SACS, the total number of blood-bags sold in the country was around six million, indicating a reporting gap of two million blood units in 2004.

**Table.** Status of blood-banking in Maharashtra and Gujarat, 2005

Indicator	Maharashtra (24)	Gujarat (25)
Population 2001 (million) (26)	96.75	50.56
Rural population (%) (26)	56	63
Number of blood-banks	273	162
Government	74	29
IRCSs	10	11
Charitable Trust∗	134	51
Private	55	71
Number of blood-banks per 100,000 people	0.26	0.32
Total blood collection (units)	874,034	513,203
Blood units per 100,000 people	903	1014
% of increase in total blood collection, 1998-2005	88.0	102.7
Total blood collection by type of blood-banks (%)		
Government	31.0	12.5
Charitable Trust∗	51.6	61.5
IRCSs	9.7	13.0
Private	7.7	13.0
Voluntary blood collection (%)	74.6	63.9
% of increase in voluntary donation, 1998-2005	56.7	89.5
Voluntary blood collection by type of blood-banks (%)		
Government	74.3	42.0
Charitable Trust∗	76.8	74.1
IRCSs	88.2	70.1
Private	43.5	30.4
Prevalence of major infections in total collected blood units		
HIV+	0.66	0.32
HBs Ag+	1.73	1.07
HCV+	0.48	0.23
VDRL+	0.25	0.25
Total safe blood (%)	96.87	98.13
Safe blood units per 100,000 people	872	995
Component separation units	96	29
% of use of blood units for component separation	40.0	38.0
Blood-donation camps	10,461	5,592

∗Charitable Trusts include voluntary blood-banks, Lions Club, Rotary Club, etc.; HBs=Hepatitis B surface antigen; HCV=Hepatitis C virus; HIV=Human immunodeficiency virus; IRCS=Indian Red Cross Society; VDRL=Venereal Disease Research Laboratory

Maharashtra has 273 registered blood-banks, many of which are managed by charitable trusts (n=134). In Gujarat, there are 162 blood-banks, of which 71 are run by private organizations. The majority of blood collection in both the states is done by the blood-banks managed by charitable trusts, followed by government blood-banks, Indian Red Cross Societies (IRCS), and private blood-banks. Charitable trust and IRCS blood-banks also contribute significantly to voluntary collection of blood in both the states. This may be because of the high level of motivation of staff, good-quality services, and promotional outreach efforts by charitable trusts and IRCS to reach voluntary blood donors, such as organizing outdoor blood-collection drive, motivating donors by public recognition, continuous contact, and motivation of blood-donor groups in various organizations.

According to the WHO, blood-banks in the government hospitals usually have low status within laboratories and are usually run by a laboratory technologist, often inappropriately trained and inadequately supervised (5). This, in turn, may affect the functioning of blood-banks. The government blood-banks in Maharashtra contribute to 31% of the total blood collection whereas, in Gujarat, it is as low as 13%. Contribution of voluntary donation of blood in the total blood collected by the government blood-banks is 74% in Maharashtra and 42% in Gujarat. The active role played by the independent State Blood Transfusion Council in Maharashtra may have contributed to the significant improvement in the status of the government blood-banks in Maharashtra.

### Blood donors

Lack of voluntary non-remunerated donors of blood is still the main constraint for blood safety in India. Replacement donors, those related to the recipient, still provide the bulk of blood for blood-banks. Some professional donors pose as relatives of the recipient and get clandestinely paid in some states where the voluntary donation movement is weak. In India, during 2004, only 55% of the total blood collection was through voluntary donation. It includes blood units from 36,855 blood-donation camps.

Voluntary blood-donation movement is relatively strong in Maharashtra and Gujarat. There was 57% increase in voluntary blood donations in Maharashtra and 89% in Gujarat between 1998 and 2005. The increase in blood donation was partly because more camps were organized by the states. In Maharashtra, the number of blood-donation camps has tripled between 1998 and 2005. It is also observed that the total blood collection has gone up in Maharashtra by 88% and in Gujarat by 103% over the same period.

About 0.9% of the population in Maharashtra donates blood whereas 1% of Gujarat population donates blood which matches the lower limit of the WHO's estimate of need for donation of blood at 1% of the population.

### Activities undertaken to increase donor base

Maharashtra conducts aggressive information, education, and communication (IEC) activities to increase awareness on blood safety and to promote repeated voluntary donation of blood. Action plans have been prepared in detail for various activities, such as mass media campaigns, blood-grouping camps, video films, cassettes, articles, messages, slogans, posters, fact sheets, and *rangoli* (a design made by coloured sand/rice-powder on the floor during Indian festivals) competitions. Registration of prospective blood donors is carried out at different public places in Mumbai, especially at railway stations. Blood-donation awareness rallies, distribution of educational materials, appreciation and felicitation of ‘centurion donors' (those who have donated 100 units of blood over a period), etc. are part of IEC activities adopted by the Maharashtra SBTC for promoting voluntary blood donation. Camp organizers, who collect more than 1,000 units of blood, are also facilitated. Efforts, such as birthday blood-donation campaigns, are put on to retain voluntary repeated blood donors. Targets given to the Primary Health Centres (PHCs) to organize blood-donation camps not only help in collecting blood but also help in spreading awareness about voluntary blood donation in rural areas. Uniform guidelines for blood-donation camps are circulated in the state. The Maharashtra SBTC organizes special camps, i.e. camps of doctors, police, etc., to create motivation and remove misconceptions.

NGOs and individuals in Gujarat are quite active in promoting voluntary donation of blood. According to the Gujarat SACS, 86% of its population is exposed to the promotional campaign for voluntary donation, the highest in the country (25). Promotion of voluntary donation of blood and creation of awareness through various media, such as print, competitions, rallies, etc., is done by individual blood-banks. An IRCS facilitates ‘centurion donors', handicapped donors, and female blood donors. The GSACS appeals to blood-banks to provide iron supplements to women visiting blood-banks to generate a donor base. Another donor base is created through the School Adolescent Education Programmes targeting 9^th^ and 10^th^ standard students while creating awareness. Children, whose parents are blood donors, are provided with badges, stating ‘My Parents are Regular Blood Donors'. To promote a repeat donor base, the blood-banks send birthday greetings and reminder-calls to donors.

### Testing and processing

Although India has regulations to screen donated blood for HIV, hepatitis B and C, malaria, and syphilis, coverage and sustainability of screening depend on the availability of testing reagents and budget for the same. One hundred percent of blood units in licensed blood-banks are to be screened for all major diseases; however, the status of screening among unregistered blood-banks is unknown. Nationally, 1.2% of blood units are positive for hepatitis B, followed by 0.48% for hepatitis C, and 0.34% for HIV. Thus, an estimated 2% of blood collected is infected and is discarded.

In Maharashtra, as all blood-banks report to the SBTC, each collected blood unit is screened. In 2005, the seropositivity of HIV in Maharashtra was 0.66%. The state achieved a decline (60.4%) in seropositivity of HIV, along with all major diseases in collected blood units between 1998 and 2005. After screening for all major diseases, 97% of colleted blood units are safe to use; the remaining 3% infected units are discarded.

In Gujarat, some blood-banks are not regularly reporting to the Gujarat SACS. Therefore, the screening status of non-reporting blood-banks is not known. In 1998, the seropositivity of HIV was 0.36%, which reduced to 0.32% in 2005, a decline of 11%. Almost 98% of the total blood collection is safe to use.

### Clinical use of blood—blood-component therapy

As reported by key officers of the major blood-banks, the inappropriate use of blood is assumed widespread as single-unit transfusion is often given which is not believed to be of much help. This includes the transfusion of blood or blood products in the presence of safer alternative therapies. With no monitoring system in place, however, it is difficult to ensure the rational use of blood. The use of blood in hospitals is hardly audited in India.

In most developed countries, 75-100% of blood is transfused as components whereas, in developing countries, most blood transfused is as whole blood. In India, only 20% of blood units have been segregated into components (27). The state of Maharashtra has 96 blood-component separation facilities segregating 40% of the total blood units into components. In Gujarat, 29 blood-banks are providing 38% blood components. The blood-banks managed by Charitable Trusts segregate the highest proportion of blood into components (46%), followed by the IRCS (24%).

Promoting blood-component therapy would not only bring down the requirement of whole blood by 60-70%, it would also ensure the optimum use of all resources invested in the blood-component separation facility. The use of blood-component therapy allows four patients to benefit from one unit of blood collected. [WHO recommends that the ratio of the use of blood components to whole blood should be 90:10, since only a limited category of clinical interventions requires whole blood (27)]. Therefore, it becomes necessary to promote blood-component therapy, which should be actively taken up by the Blood Transfusion Councils.

### Quality system

Given the overall negligence to blood-banking in India, it is not surprising that its quality control has not been seen as an essential part. A lack of quality-assurance measures, including manuals of standard operating procedures, appropriate training and competency certification programmes, and continuous assessment systems, often hinders the implementation of good laboratory and manufacturing practices. For example, in Gujarat, some stakeholders reported that different blood-banks use different testing procedures and testing standards which hinders the exchange of blood between blood-banks. This may lead to more wastage of blood.

### Recent blood-bank modernization scheme of NACO

Under the Blood Bank Modernization scheme of NACO, till November 2006, the Government of India has provided support to modernize 883 district-level blood-banks and 255 major blood-banks and established 82 blood-component separation units in the country. Ten state-of-the-art blood-banks were also established (28). The Maharashtra state has upgraded 31 major blood-banks and 41 district-level blood-banks. A large metropolitan blood-bank is being established in the city of Mumbai, with an estimated annual voluntary collection of 50,000 units at a cost of Rs 50 million. In Gujarat, under the programme, there are 15 major blood-banks and 42 district-level blood-banks.

### Training

Maharashtra conducts regular training programmes on various issues to strengthen the capacities of professionals engaged in blood-banking services. The training programmes cover the following:

• Modular training for all blood-bank personnel
• Training on hepatitis C, ELISA Reader maintenance and repair, biosafety measures for all zonal BTC Blood Transfusion Officers and Technicians
• Component preparation for Blood Transfusion Officers and Technicians
• Blood group serology for Blood Transfusion Officers and Technician at the Indian Council of Medical Research
• Genetic blood disorder detection for Blood Transfusion Officers and Technicians
• Training for Drug Inspectors
• Quality-management training—Blood Transfusion Officers
• Transfusion and transplantation science
• Key Blood Transfusion Officers from medical colleges trained as key trainers to undertake training for technicians of the FRU.

In Gujarat, training programmes for blood-bank personnel are offered for updating their skills and knowledge. The training programmes, usually offered once in three months in various regions of the State, usually address topics on necessity of safe blood, the rational use of blood, and clinical procedures.

Recently, the Medical Council of India (National Education Regulatory Board) has approved a postgraduate course in blood-banking and immunology haematology/immunology haematology and blood transfusion. Presently, five medical colleges offer this postgraduate course in the states of Gujarat, Jammu and Kashmir, Maharashtra, Tamil Nadu, and Uttar Pradesh (29).

### Monitoring

Maharashtra maintains a reasonably good computerized management information system (MIS). Reports are compiled at the state level by the 5^th^ of every month, and feedback is given to the regional health officers by the 15^th^ of every month. At the start of each year, monthly analysis of blood collection of the previous year is done to formulate strategies to compensate for the deficit in blood collection in a particular month. Maharashtra has also developed an excellent system of web-based monitoring of storage and availability of blood units and components of each blood group in every blood-bank in the state. This allows hospitals and blood-banks to request blood from neighbouring blood-banks where the needed group may be available. This ensures the most efficient use of available blood and minimizes wastage.

Gujarat, unfortunately, does not have a similar system. Hence, in the case of need, either blood-bank managers have to call the other blood-banks to check the availability or the relatives of the patient have to visit more than one blood-bank to procure required type of blood. For monitoring the blood-banks, the state maintains a regular database for all the reporting blood-banks. However, feedback is only occasionally provided to blood-banks by the GSACS.

### Supply and access to safe blood

Blood is urgently required for saving women's lives in the case of massive postpartum or antepartum haemorrhage or severe anaemia. As the majority of blood-banks are located in urban areas, the availability of blood in rural areas is difficult. To address this lack of availability of blood in rural areas, the policy was changed in 2001, allowing the establishment of blood-storage units in rural regions. However, nothing much happened on this front as it was given low priority in the states. In 2005, the National Rural Health Mission dedicated a part of its funds for the establishment of blood-storage units at the FRUs. These blood-storage units will be attached to large regional blood-banks in urban areas for blood supply.

In Maharashtra, 44 blood-storage centres are currently functional in the FRUs. By the end of March 2009, the target is to open 171 blood-storage units. By 2012, 400 blood-storage units will be functional in rural hospitals. Under a recent amendment (2006) of the Nursing Home Registration Act of Maharashtra, all nursing homes, clinics, and private hospitals should be affiliated with a licensed blood-bank for procurement of blood for patients admitted to their hospitals. Along with this, the state also requires blood-bank or storage facility within five km of any registered private abortion facility to ensure the availability of safe blood for transfusion services, if required (30). All registered medical facilities have access to the website information system of Maharashtra SBTC described above and can book required blood units in advance before surgery. The facility helps nursing homes obtain blood products in emergency, without wasting time.

All blood-banks and blood-storage centres have to maintain a record on blood collection and distribution in Maharashtra. All regional blood-banks supplying blood units to blood-storage centres record the information on the number of blood units supplied, blood group of the supplied blood units, and dates of issue and expiry. The storage centres and private hospitals/clinics also maintain information on similar indicators to avoid wastage of blood units, as unused blood units can be returned to the regional blood-bank before the date of expiry. However, it has been observed that the government centres discard units after the expiry as there is not much accountability relating to this. The institutions also maintain the records of distribution of blood units, with the name of the patient and hospital, blood group of the patient, and units of blood given. However, this information is not analyzed for management purpose in Maharashtra.

The below poverty-line (BPL) population is entitled to receive blood and blood products free of charge from any government blood-bank or storage centre in Maharashtra. Individuals affected by thalassaemia, haemophilia, and sickle-cell anaemia also receive blood free of charge on their identity card. Approximately 40,000 units of blood are given to thalassaemia patients every year in Maharashtra. All voluntary donors of blood are also entitled to one free unit of blood on production of the voluntary blood donor card.

The Gujarat State AIDS Control Society arranged a meeting to discuss the “Reorganization of blood-transfusion services and developing blood-storage centres at the FRUs” at the end of 2005 to make safe blood available. Recent data on blood-storage units by the Government of Gujarat showed that 26 of the 39 functional blood-storage units in Gujarat are attached to the FRUs required in the state. The blood-storage units are located in the FRUs attached to charitable hospitals, community health centres, and district hospitals. All blood-storage centres are linked to the RBTCs for blood supply. The Government of Gujarat is also planning to recognize more blood-banks as RBTCs to strengthen blood supply in rural areas. Recently, the Gujarat State AIDS Control Society has initiated a free blood scheme for thalassaemia, haemophilia, and sickle-cell patients.

By law, there is no charge for blood but blood-processing and testing cost is generally allowed. The charge for blood-transfusion service in the government blood-banks is only Rs 425 to any patient (approximately US$ 9.4). The cost in private blood-banks varies from centre to centre and is generally higher.

## CONCLUSION AND RECOMMENDATIONS

While the Indian health sector has made some noteworthy achievements over the last 50 years, it has not responded satisfactorily enough to meet the national goals on blood-transfusion services as is witnessed by the substantial negligence to blood-banking services in the country. In India, blood transfusion relies on very fragmented blood-supply systems, where control is exercised by different layers of the Government, making it difficult to assure the quality of blood and blood products. Two parallel systems are in place to monitor the blood-safety programme in India—NBTC/SBTC and NACO/SACS.

Given the fact that there are a large number of Charitable Trusts and independent commercial and private blood-banks, the control of quality and coordination of all blood-banks to ensure easy access to blood and minimize wastage are a challenging task. This requires much more managerial capacity, skills, and resources within the State Blood Transfusion Councils. The promotion of postgraduate courses in blood-transfusion services will help in getting the trained and skilled human resource in the field of blood-banking. Given the lack of government resources in blood-transfusion services, there has been poor quality of service and periodic shortages of blood. Many blood-banks depend on philanthropic funding for their basic operations. The Government needs to increase its commitment towards improvement of blood-banking services and ensure that all needy patients get blood on time and free of charge for the BPL population.

The existing MIS for blood-banking seems weak although it has been improved in Maharashtra. There is a lack of proper records on the use of blood in India. Due to this, it is not possible to estimate the need, supply, and unmet need for blood in obstetric emergency. Besides, the monitoring of the clinical use of blood and blood products is weak. No systematic analysis is done to measure adverse reactions following blood transfusion. Blood-banks also lack registering or monitoring of the total demand for blood and blood products (requests received) for each blood group that could not be met due to the non-availability of blood or blood products. They only record blood collected and used. They also do not report systematically the proportion of stored blood that expired due to non-use. Thus, substantial improvement in the MIS is needed.

We find that, although the overall availability of blood is almost the same in both the states, the organization of blood-transfusion services is much better in Maharashtra as the Maharashtra Blood Transfusion Council is an independent unit with full-time staff under active leadership. Learning from this experience of Maharashtra, we recommend all the states to have independent and well-staffed blood councils. In Gujarat, the NGOs and the Indian Red Cross Societies are actively promoting voluntary donation of blood and, hence, per-capita collection in Gujarat is slightly better than in Maharashtra. Learning from Gujarat indicates the possibility of the dynamic role being played by the NGOs and IRCS in blood-banking.

Ensuring a safe supply of blood and blood products and the appropriate and rational clinical use of blood are important public-health responsibilities of every national and state government, especially for saving lives of mothers who need comprehensive EmOC services because of pregnancy-related haemorrhage, severe anaemia, or abortions. The mechanism of availability of blood in rural areas is quite frail. There is a need for networking between urban blood-banks and rural blood-storage units. And as there is no special provision for timely blood supply for maternal emergencies in rural areas, it may not be possible to reduce maternal mortality due to haemorrhage, anaemia, and abortions. The use of blood components, at least in urban areas where facilities exist for storage of components, will also enhance supply of whole blood for rural areas.

## ACKNOWLEDGEMENTS

The authors acknowledge the International Centre for Diarrhoeal Disease Research, Bangladesh, Department of International Development (DFID), United Kingdom, and GSACS for partial funding for the study. They are also thankful to the Maharashtra State Blood Transfusion Council and the Gujarat State AIDS Control Society for their valuable contributions and whole-hearted cooperation in carrying out this study.

Its content are solely the responsibility of the authors and do not necessarily represent the official views of the DFID.
